# Penthrox Is an Effective Analgesic but Is It Patient Approved?

**DOI:** 10.7759/cureus.53537

**Published:** 2024-02-04

**Authors:** Ana V Dias, Ziad Zeidan, Matt Copp, Frances Eslabra, Rawan Hassan, Rory Middleton

**Affiliations:** 1 Orthopaedics, Royal College of Surgeons of England, London, GBR; 2 Orthopaedics and Trauma, Royal Cornwall Hospital, Truro, GBR; 3 Orthopaedics, Royal Cornwall Hospital, Truro, GBR

**Keywords:** patient feedback, patient controlled analgesia, orthopaedics trauma, trauma, manipulation under anesthesia

## Abstract

Background

Penthrox is a handheld inhaler that administers methoxyflurane. Its use is approved for analgesia in moderate-to-severe trauma-related pain in adults in the ED. The literature currently lacks methodologically robust qualitative data on individual patient experiences. Using a structured qualitative study, we set out to address this shortcoming.

Methods

Five patients were selected as a focus group to identify key themes they felt were important to explore, and these were included in the questionnaire design. We retrospectively identified all uses of Penthrox in the ED from June to August 2021. Qualitative data was gathered using the Trickett short interview method, and responses were grouped into positive and negative descriptors. In addition, quantitative data concerning their experience using the 5-point Likert scale was also gathered.

Results

A total of 101 participants responded to the questionnaire. Penthrox was utilised mainly for the manipulation of fractures, most commonly those of the ankle and wrist. Around 90% reported an overall satisfaction of ≥ good, and 97% reported the ease of use to be ≥ good. Its analgesic effectiveness was rated as excellent by 52%, and ≥ good by 89%. The most reported side effects were drowsiness (13%) and nausea (7%). The majority reported no side effects (74%). About 94% of the participants said they would take it again if required. An NVivo word cloud (Lumivero, Denver, CO, USA) was created visually, confirming an overall positive experience amongst the patients.

Conclusions

This study shows that Penthrox is a well-tolerated and user-friendly means of alleviating trauma-related pain in the ED. It highlights the importance of taking into consideration the individual patient journey alongside robust evidence-based data on safety and efficacy for the development of a holistic treatment.

## Introduction

Penthrox is a handheld inhaler that administers a low dose of methoxyflurane [1]. It was first used as a general anaesthetic in the 1960s, but concerns about its nephrotoxicity made it obsolete [1]. However, at low doses, it is an effective analgesic with a negligible toxicity profile [1], which affects many ion channels and receptors at gap junctions, causing slight muscle relaxation and pain relief. Since being approved in Europe in 2015, Penthrox has been used for analgesia in moderate-to-severe trauma-related pain in adults in the ED. Despite being widely used in Australia, it is still not commonly used in the United Kingdom [1]. During the COVID-19 pandemic, patients needed to be treated as efficiently as possible and limit admission to the hospital to save resources for the new demand, i.e., COVID-19. To decrease the need to admit patients and utilise operating theatres, Penthrox was adopted as an analgesic to facilitate the management of fractures, joint dislocations, and traumatic wound care in the ED. A previous local study carried out at our institution showed that Penthrox is a safe and effective tool that facilitates a reduced length of hospital stay for patients [2]. This study, however, did not consider the individual patient experience.

Recently, there has been increased scientific debate in the literature regarding utilising patient experiences in healthcare decision-making, both on a local and national level [3-5]. The current school of thought is that patients should be viewed as stakeholders in the treatments they receive, and therefore, their input should be considered in decisions regarding them [3,6]. The traditional model for decision-making is based largely on objective data related to efficacy, safety, and cost-effectiveness, without considering subjective data on the patient experience [3]. Nevertheless, studies in the literature have shown that, with methodologically robust research, engaging patients and using their experiences as evidence serves to enhance the likelihood of patients engaging with the proposed treatment [3,6,7]. Furthermore, by understanding the nature of living with a certain condition or ailment, clinical decision-making or decisions on resource allocation can be made more accurately [3]. This is especially effective when the clinical efficacy of a treatment is not yet known, for instance, when the treatment is in its early stages or when there is a small subset of patients accessing the treatment [8].

In the field of orthopaedics, assessing patient perspectives has been utilised to good effect in establishing a video consent tool in addition to traditional methods for spinal surgery [9]. In this study, researchers assessed patients’ satisfaction and feedback on the video consent tool, which improved its uptake when it was eventually established [9]. Furthermore, the assessment of the patient experience postoperatively in one American study allowed clinicians to identify factors that could be optimised to improve patient care [10]. The accelerated use of Penthrox at our hospital was brought about by the COVID-19 pandemic, as were remote consultations, which have been assessed in a British tertiary orthopaedic rehabilitation centre [11]. This study identified means of aligning patient and clinician preferences with the use of remote consultations and relied upon evidence gathered from the patient experience [11]. Thereby, highlighting the increasing role of studies assessing the patient experience in various aspects related to orthopaedic surgery. At the time of writing, it was noted that there were no studies in the literature related to the patient experience using Penthrox for trauma-related pain.

The key issue noted in relation to studies pertaining to the patient experience relates to the methodology; however, the literature highlights the importance of extracting qualitative data in a reliable fashion [3]. The scientific literature currently lacks methodologically robust qualitative data on the individual patient experience relating to the use of Penthrox for orthopaedic procedures in the ED. Using a structured qualitative study based on available evidence in the literature, we set out to address this shortcoming.

This study was previously presented as a talking poster at the Association of Surgeons of Great Britain and Ireland (ASGBI) Reset and Recharge Congress on May 4th, 2022; the Association of Surgeons in Training (AsiT) 47th Annual Congress on March 4th and 5th, 2023; and the Royal College of Surgeons of Edinburgh (RCSEd) Annual QI and Audit Symposium on April 28th, 2023. It was also presented as an oral presentation at the International Confederation of Plastic Surgery Societies (ICOPLAST) 2023 International Conference on May 5, 2023; the ASGBI Congress 2023 on May 19, 2023; and the British Trauma Society (BTS) Annual Scientific Meeting 2023 on November 23, 2023.

## Materials and methods

We retrospectively identified all uses of Penthrox in the Royal Cornwall Hospital ED from December 2020 to August 2021. During this timeframe, 219 patients were identified in the records as having received Penthrox. Five patients who had received Penthrox were randomly selected using a random number generator to identify key themes they felt were important to explore. These themes were then used to form an essential part of the questionnaire design. The merits of utilising a focus group in qualitative research are that it allows the research participants to generate their questions [12]. When utilised alongside clinicians’ qualitative and quantitative data, it allows for the research participants to have acted as stakeholders in the project's development [12].

The focus group highlighted the importance of looking into the user-friendliness of the device, analgesic effectiveness, and side effects. They reflected a positive response to the study and were grateful for the consideration of their perspective. Their insight was taken into account to design the questionnaire used in the study (Table 1).

**Table 1:** Patient questionnaire

No.	Questions
1	What orthopaedic procedure was undertaken at the emergency department?
2	Tell me about your experience
3	How many boxes of Penthrox were used?
4	How would you rate your overall satisfaction after using the Penthrox device? Was it excellent/very good/good/fair or poor?
5	How good was the explanation of how to use the Penthrox device? Was it excellent/very good/good/fair or poor?
6	How would you rate the patient-friendliness of the Penthrox device? Was it excellent/very good/good/fair or poor?
7	How effective was Penthrox at reducing pain during your procedure? Was it excellent/very good/good/fair or poor?
8	Did you experience any adverse events? If so, what were they?
9	Were you admitted to the hospital? If so, did you have an operation?
10	Would you consider taking Penthrox again?
11	How could we have made your experience better?
12	Do you have any final comments?

Study design  

Electronic medical records were utilised to determine the indication for the Penthrox prescription. Qualitative data was gathered using the Trickett short interview method, and responses were grouped into positive and negative descriptors. The interview included five focused questions and three open questions. The focused questions also incorporated 5-point Likert scales to gather quantitative data, where potential responses were 'excellent', 'very good’, ‘good’, 'fair', and ‘poor’. An NVivo word cloud (Lumivero, Denver, CO, USA) was utilised to capture the overall patient experience. During data collection, 101 patients consented and were interviewed via telephone. The remaining 118 patients either could not be contacted or did not consent to the interview.

## Results

**Figure 1:**
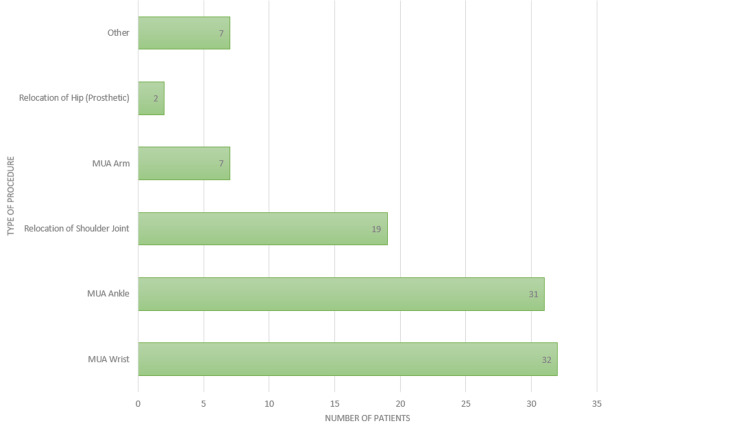
Bar chart displaying the frequency of procedures for which Penthrox was utilised during the study period (n = 101) MUA: Manipulation under anaesthesia

**Figure 2:**
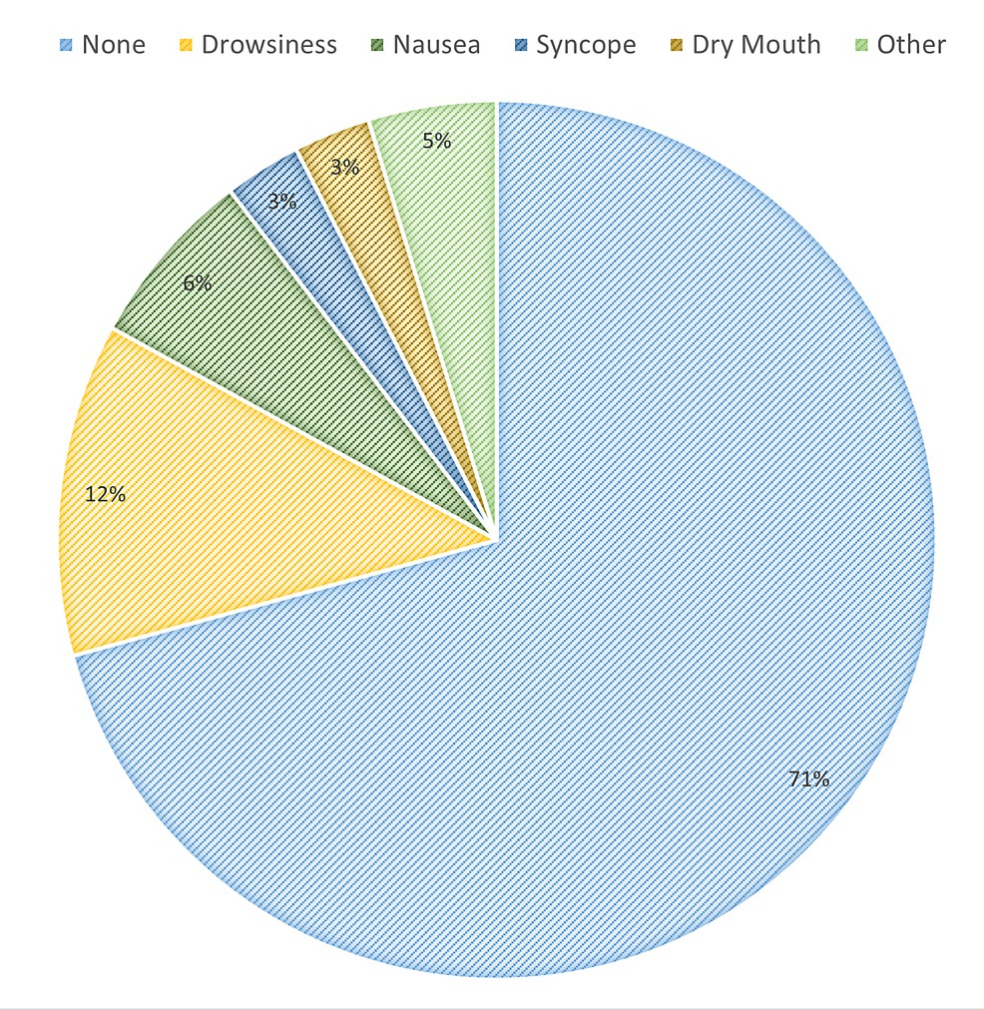
Pie chart displaying the side effects reported from using Penthrox during the study period

**Figure 3:**
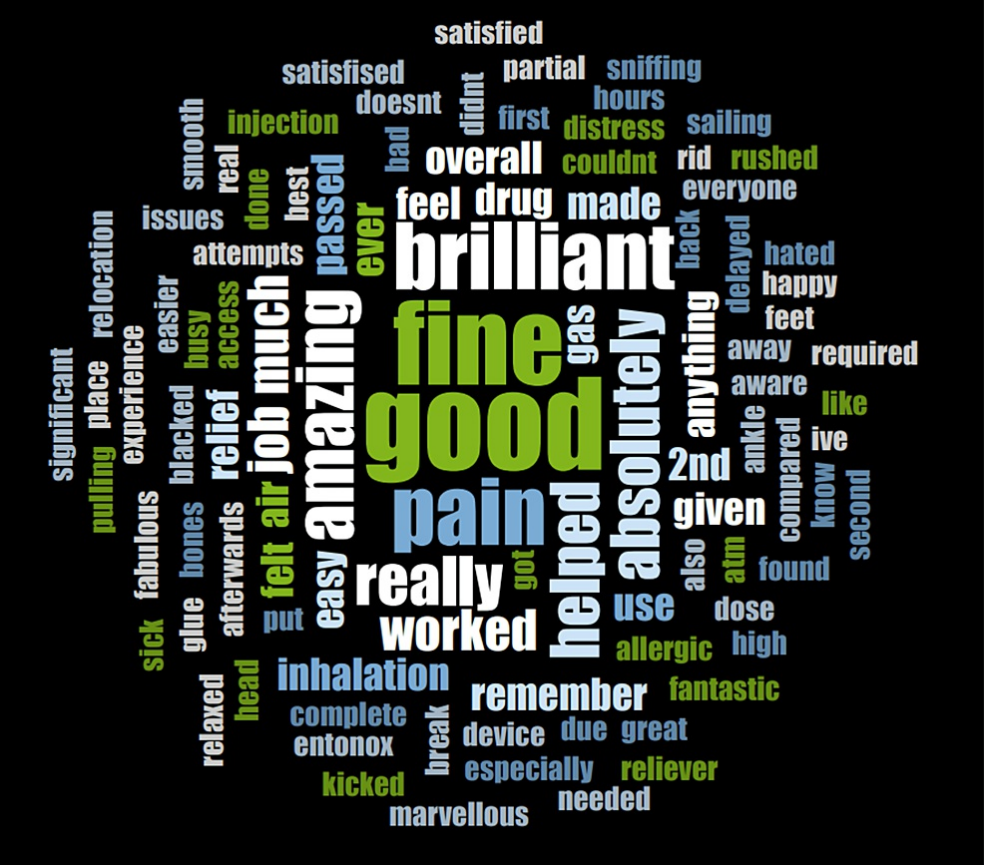
The NVivo word cloud created from responses to the open question, 'Tell me about your experience'. The pattern of responses confers an overall positive experience using Penthrox.

Out of 101 patients who consented to an interview, 61 were female and 40 were male. The median age of the patients was 52.9 years (SD = 18.58); the youngest patient interviewed was 20 years old, and the oldest was 89 years old. Penthrox was used mainly for the manipulation of fractures, particularly of the ankle and wrist (Figure 1). In terms of overall satisfaction with the Penthrox device, 90.1% (n = 91) reported an overall satisfaction of ≥ good (Table 2). Meanwhile, 96.0% (n = 97) of respondents rated the explanation of how to use the device as ≥ good (Table 2). Around 97.0% (n = 98) reported the user-friendliness of the device as ≥ good (Table 2). Furthermore, its analgesic effectiveness was rated as excellent by 52.5% (n = 52), and ≥ good by 89.1% (n = 90) of patients (Table 2). About 90.9% (n = 91) of patients only required one dose of Penthrox, while 9.9% (n = 10) of patients required two. The most reported side effects were drowsiness (12.8%, n = 13) and nausea (6.9%, n = 7). However, the majority (74.3%, n = 75) reported no side effects (Figure 2). Around 94.1% (n = 95) of patients reported they would use Penthrox again if required. The identified factors that likely contributed to the reluctance to use Penthrox again included side effects (n = 4), poor analgesic effect (n = 4), poor patient friendliness (n = 2), poor explanation (n = 2), and premature manipulation (n = 1). While 85.1% (n = 86) of patients suggested no potential areas of improvement needed, 9.9% (n = 10) suggested earlier administration, 2.0% (n = 2) implied they wanted alternative analgesia, 1.0% (n = 1) suggested an improved explanation of side effects, 1.0% (n = 1) wanted no side effects, and 1.0% (n = 1) specified delayed manipulation. The NVivo software was used to create a word cloud from patients’ responses to question 12, visually confirming an overall positive experience (Figure 3). Eleven patients independently drew comparisons with Entonox: seven patients stated a preference for Penthrox, two stated a preference for Entonox, and two did not state a preference.

**Table 2:** Quantitative data gathered from four focused questions during the interview

Question	Excellent	≥ Good
How satisfied are you overall with the Penthrox device?	54%	90%
How good was the explanation provided by the clinician?	52%	97%
How user-friendly would you rate the Penthrox device?	55%	73%
How effective was Penthrox at reducing your pain during your procedure?	52%	89%

## Discussion

A few studies exist in the literature that have investigated the efficacy and safety profile of Penthrox in the ED, the most notable being the UK-based STOP trial. This was a multi-centre, double-blind, placebo-controlled study investigating the efficacy of Penthrox in patients presenting with minor trauma to the ED [1]. It demonstrated that Penthrox had a highly significant change in pain score compared to placebo, which was most notable at 15 minutes following administration [1]. However, the median time demonstrated to achieve an analgesic effect was 4 minutes [1]. In addition, the Methoxyflurane in Emergency Department in Italy (MEDITA) study displayed the superiority of Penthrox to other forms of analgesia, such as intravenous morphine [13]. On a regional level, our previous study also showed Penthrox to be a safe, rapid, and efficacious analgesic for the management of fractures, joint dislocations, and various minor procedures in trauma patients [2]. Furthermore, it showed that the use of Penthrox in the ED was associated with a reduced length of stay in the hospital [2]. This, however, is the first study that has focused on patients’ individual experiences using Penthrox. Autonomy stands as a core ethical pillar of medicine, and as we move from a paternalistic approach to that of shared decision-making, patient experience studies increase in importance. Investigating patients’ needs and how to meet them is, therefore, imperative [3]. To achieve this, patients need to be heard and their experiences highlighted.

Optimising patient journeys through EDs is crucial, and early treatment access is a key area for improvement. The Welsh Ambulance Services NHS Trust has nationally adopted Penthrox for prehospital use, enhancing patient experience in the ED [14]. A study by the East Midlands Ambulance Service (EMAS) NHS Trust revealed that Penthrox offers a quicker and more significant analgesic response compared to other analgesics, albeit at a higher economic cost [15]. While Penthrox offers benefits over Entonox, it incurs an additional cost of approximately £12.30 per patient, per their cost-benefit analysis [15]. Despite these benefits, there is ambiguity regarding the frequency of Penthrox administration in a day. The British National Formulary specifies only a maximum weekly dose, leading to uncertainty about whether patients can receive more than one dose in the ED if they have already been administered prehospital Penthrox [16]. Since 9.9% (n = 10) of patients require a second dose, this limitation necessitates considering alternative analgesics like haematoma blocks or propofol sedation earlier in some cases.

Furthermore, there is a need for improved healthcare professional training in Penthrox usage, particularly in explaining side effects and delaying manipulation until analgesia onset. To enhance patient awareness of potential side effects, the provision of small patient leaflets during the prescription and preparation of the device is recommended. Such educational enhancements could increase patient understanding and acceptance of Penthrox, addressing its underutilisation due to concerns about side effects.

It is unsurprising that patients draw similarities between Penthrox and Entonox, given that they are both inhaled analgesics frequently used in prehospital injury and trauma. While demonstrated to be a superior analgesic in the prehospital setting [15], we were not able to find literature demonstrating this effect in the ED. Neither were we able to find evidence comparing patient experiences pertaining to these analgesics. Penthrox has a lower carbon dioxide equivalent compared to Entonox (0.84 kg CO2e vs. 98.89 kg CO2e, respectively), resulting in a lower greenhouse effect per use [17]. In the context of the NHS' efforts to reduce its carbon footprint, future research comparing patient opinions and experiences with Penthrox and Entonox could align with NHS goals.

The administration of medical treatments is a significant event, impacts patients both physically and mentally. Most existing studies prioritise objective measures such as treatment efficacy and side effects. However, discussions with a focus group emphasised the importance of qualitative studies in understanding patient perspectives, especially in the context of acute trauma pain, which is challenging to quantify. Therefore, cost-effectiveness studies used for broader cost-benefit analyses should incorporate variations in patient experience, as investigated in this study. This approach would not only address economic concerns but also enhance patient-centered care within the NHS framework.

Limitations  

The main limitation of this study is its inability to reach a significant number of patients via telephone, which results in non-responder bias. This may have skewed our findings and limited their applicability to a broader patient population. Future research should employ more effective patient contact strategies, potentially incorporating alternative means of communication beyond telephone calls. An analysis of the demographics of persons not reached could also be done to identify which populations are not represented and therefore cannot be generalized to and to inform contact technique selection for future research.

In future studies, addressing the limitations of recall bias observed in this retrospective study is crucial. It is beneficial to conduct interviews closer to the event of the ED visit. This approach could ensure more accurate and reliable data collection, as the details of the patient's experience and the effects of Penthrox on their alertness are likely to be better remembered shortly after the event.

Future studies should also consider a more controlled design to accurately assess Penthrox's effectiveness and side effects in the ED. The concurrent use of other analgesics in this study, prior to administering Penthrox, may have confounded the assessment of its true analgesic response and side effects. Although this reflects the real-world scenario where Penthrox is not used in isolation, it compromises the ability to isolate its specific impact. While this study did not aim to directly compare Penthrox with other analgesics, the inclusion of such a control group could provide valuable insights into its specific relative efficacy and patient acceptability. 

## Conclusions

This retrospective qualitative study demonstrates that Penthrox is a well-tolerated and user-friendly means of alleviating trauma-related pain in the ED. Furthermore, it provides insight into patients’ experiences using Penthrox, highlighting an overall positive experience and acceptance by patients. Further research should aim to compare patient experiences of medications commonly used for trauma-related pain (e.g., intravenous morphine and Entonox) with Penthrox. This will allow for a greater understanding of which analgesic is most suitable for this indication. Moving forward, this study has shown the importance of considering the individual patient journey alongside robust evidence-based data on safety and efficacy for the development of a holistic treatment to ensure it is accepted by those using it.
